# Region-Specific Lipid Alterations Around the 28-Year Transition as Early Indicators of Skin Aging

**DOI:** 10.3390/metabo16010073

**Published:** 2026-01-13

**Authors:** Meiting Yi, Qian Jiao, Jianbiao He, Huiliang Li, Yangyang Fang, Youjie He, Huaming He, Yan Jia

**Affiliations:** 1 Key Laboratory of Cosmetics of China National Light Industry, College of Light Industry Science and Engineering, Beijing Technology and Business University, Beijing 100048, China; wjmtoct03@163.com (M.Y.); 18210788848@163.com (Q.J.); 18813061209@163.com (J.H.); 2 Hangzhou Huaningxiang Biotechnology Co., Ltd., Hangzhou 310000, China; yuanzhi@eastgarden.com (H.L.); pangyang@eastgarden.com (Y.F.); 3 College of Biomass Science and Engineering, Sichuan University, Chengdu 610000, China; heyoujie@scu.edu.cn; 4 Academy for Interdisciplinary Studies, College of Light Industry Science and Engineering, Beijing Technology and Business University, Beijing 100048, China

**Keywords:** skin aging, skin surface lipids, combination skin, skin lipidomics

## Abstract

**Background**: Early molecular changes on the facial skin surface during early adulthood remain insufficiently characterized. We integrated biophysical readouts with untargeted skin surface lipid (SSL) profiling to identify region-dependent, age-associated features in women with combination skin. **Methods**: Eighty healthy Chinese women were stratified into 22–28 years (*n* = 40) and 29–35 years (*n* = 40). Sebum was measured on the cheek and forehead; cheek elasticity, hydration (CM), transepidermal water loss (TEWL), pH, and tone indices were assessed under standardized conditions. SSLs from both regions were profiled by UPLC–QTOF–MS. Differential features were prioritized using OPLS-DA (VIP > 1) with univariate screening (*p* < 0.05; fold change > 2 or <0.5). **Results**: TEWL, CM, and pH were comparable between age groups, whereas the older group showed lower cheek elasticity and reduced sebum. Lipidomics revealed clearer remodeling on the cheek than the forehead: 30 and 59 differential SSL features were identified in the cheek and forehead, respectively. Cheek changes in the older group were characterized by lower ceramides (including acylceramides), TG/DG and long-chain fatty acids, alongside relatively higher cholesteryl esters. **Conclusions**: Conventional barrier indices remained largely stable across this age window, while cheek SSL profiles captured earlier molecular shifts, providing candidates for targeted validation and longitudinal follow-up.

## 1. Introduction

Skin aging manifests as wrinkles, reduced elasticity, epidermal thinning, and barrier dysfunction due to progressive physiological decline [1,2,3]. Longitudinal data from Chinese women indicate that signs of skin aging become more pronounced between the ages of 24 and 30, with changes being more evident in the cheek region than in the forehead [4]. In traditional Chinese medical texts, a “seven-year cycle” is used as a cultural framework to describe stage-like changes across the female lifespan [5,6]. Here, we treat this concept as a culturally inspired hypothesis driver rather than biological evidence and ask whether modern biophysical readouts and SSL lipidomics can detect measurable shifts in the late twenties in women with combination skin.

With advancing age, the skin barrier weakens, shown by lower moisture retention and a higher risk of infection. Since skin lipids (like ceramides, cholesterol, and fatty acids) are key for barrier function, their significant decline during aging impairs the skin barrier’s structure and function [7]. Specific lipid metabolism genes (e.g., AGPAT1, AGPAT3, ELOVL5, AWAT2, FA2H, and FADS2) are activated to a greater extent in individuals who respond more quickly to non-ablative fractional laser (NAFL) treatment, aiding in restoring and maintaining barrier function [8]. Thus, modulating lipid metabolism is vital for enhancing skin-barrier function and slowing skin aging. Skin surface lipids (SSLs), comprising sebaceous and intercellular components, are essential for water retention, structural cohesion, and antimicrobial defense [9]. Studying the changes in SSLs during aging helps understand the molecular mechanisms of skin aging.

Combination skin, the most common facial skin type, exhibits distinct oil-dry zoning, with the T-zone (forehead, nose, chin) being lipid-rich and the U-zone (cheeks) more susceptible to dryness [10]. The spatial SSLs heterogeneity across facial zones and age-related SSLs alteration in combination skin populations are still not fully understood.

Lipidomics, a mass-spectrometry-based technique, enables high-throughput profiling of lipid classes and molecular species to reveal metabolic alterations [11]. It has been widely used to study skin lipid changes in diseases such as atopic dermatitis [12], acne [13], and rosacea [14]. In this study, Chinese women aged 22–35 years with combination skin were divided into two groups (22–28 and 29–35 years) based on questionnaire results. Through analyzing skin physiological parameters and SSL profiles, we explored how skin changes with age. Therefore, we integrated facial biophysical parameters with untargeted SSL lipidomics in Chinese women with combination skin to characterize region-dependent, age-associated lipid features during early adulthood, and to generate candidates for targeted validation and longitudinal follow-up. The TCM ‘seven-year cycle’ is cited here as cultural context rather than mechanistic evidence. These findings may inform region-targeted lipid-supportive skincare strategies (e.g., cheek-focused barrier lipid replenishment) before overt changes in TEWL or hydration become detectable.

## 2. Materials and Methods

### 2.1. Chemicals and Reagents

Ammonium formate, acetonitrile, isopropanol, methanol, formic acid, and water (LC–MS grade; Thermo Fisher Scientific, Waltham, MA, USA) were used for LC–MS analysis. Sebutape^®^ for skin surface lipid adsorption tape was purchased from CuDerm Corporation (Dallas, TX, USA).

### 2.2. Study Subjects

Eighty healthy Chinese women with combination skin were recruited using a validated self-administered questionnaire. Combination skin was defined according to the questionnaire criteria [15], reflecting oilier T-zone characteristics with relatively drier cheek/U-zone features. Participants were stratified into two age groups: 22–28 years (Y group; *n* = 40, mean age 24.16 ± 1.46) and 29–35 years (O group; *n* = 40, mean age 32.39 ± 1.92). Exclusion criteria included active dermatological conditions, recent facial medication use, light therapy exposure, smoking, or alcohol consumption. All participants provided informed consent. The sample size (*n* = 40 per age group) was determined based on feasibility and to be broadly consistent with cohort sizes commonly used in facial skin phenotyping and omics studies. No formal a priori power calculation was performed due to the exploratory nature of untargeted lipidomics.

### 2.3. Sample Collection and Preparation

Participants were assigned to Y or O groups based on age. After facial cleansing, subjects acclimated for 30 min under controlled conditions (21 ± 2 °C, 50 ± 10% RH). Skin measurements were taken on the left cheek using standardized instruments: stratum corneum hydration (Corneometer CM825), transepidermal water loss (TEWL; Tewameter TM300), skin surface pH (pH905), skin tone parameters (a*, b*, L*, ITA°; CL400), and elasticity (MPA580; parameters R2 and Q1) were measured using instruments from Courage + Khazaka electronic GmbH (Köln, Germany). Sebum levels were measured at the cheek (“C”) and forehead (“F”) using SM815. Skin surface lipids (SSLs) were collected using Sebutape^®^ patches and stored at −80 °C. All measurements were conducted under controlled temperature and humidity following the above standardization procedures.

Lipid extraction was performed using a modified Bligh–Dyer method, which was optimized in our laboratory to improve recovery of low-abundance surface lipids. Prior to UPLC–QTof–MS analysis, a pooled quality control (QC) sample—prepared by combining equal aliquots from all extracts—was analyzed at regular intervals to monitor instrumental stability.

### 2.4. Instrument Parameters

Chromatographic separation was performed on a Waters UPLC system using a CSH C18 column (1.7 μm, 2.1 × 50 mm). The mobile phases were: A, acetonitrile/water (40:60, *v*/*v*) with 0.1% formic acid and 10 mM ammonium formate; B, acetonitrile/isopropanol (90:10, *v*/*v*) with identical additives. The flow rate was 0.4 mL/min, injection volume 2 μL, and column temperature 50 °C.

Mass spectrometry was conducted using a Xevo G2-XS QTof mass spectrometer (Waters Corporation, Milford, MA, USA) equipped with an electrospray ionization (ESI) source in positive ion mode. Full-scan spectra were acquired over *m*/*z* 50–1200, with leucine enkephalin (*m*/*z* 554.2771) as the lock mass. Data were processed using MassLynx v4.1. (Waters Corporation, Milford, MA, USA).

### 2.5. Data Acquisition and Analysis Methods

Raw LC–MS data were processed in Progenesis QI v2.0 (Waters Corporation, Milford, MA, USA) for peak picking, alignment, and deconvolution, generating a feature table (*m*/*z*–retention time pairs with peak intensities). Features were normalized across samples using Progenesis global normalization and log-transformed where appropriate prior to statistical modeling. Missing values were handled by removing features with >50% missing, imputing the remaining missing values with half of the minimum positive value. Instrument stability was monitored using pooled QC injections; features with poor repeatability in QC (RSD/CV > 30%) were excluded from downstream analyses.

For biophysical parameters, two-group comparisons were performed using unpaired *t*-tests or non-parametric tests when distributional assumptions were violated. For lipidomic multivariate modeling, orthogonal partial least squares discriminant analysis (OPLS-DA) was performed in EZinfo v3.0 (Umetrics AB, Umeå, Sweden), and permutation testing was used to assess model robustness. variable importance in projection (VIP)-selected differential features were defined as VIP > 1 together with univariate *p* < 0.05 and fold change > 2 or <0.5. For analyses involving both age group and facial region, linear mixed-effects models were fitted with age group, region and their interaction as fixed effects and subject as a random intercept; post hoc contrasts were adjusted using the Holm method. Linear mixed-effects models were also used to assess continuous age trends. Pearson correlation was used for lipid–biophysical associations. For reaction-chain mapping, differential features were carried forward using the same exploratory criteria as above (VIP > 1 with unadjusted *p* < 0.05) to maximize network coverage. Effect estimates with 95% confidence intervals for the planned contrasts and the region-specific age slopes are provided in Supplementary Table S2.

## 3. Results

### 3.1. Age-Related Changes in Skin Physiological Parameters

**Figure 1:**
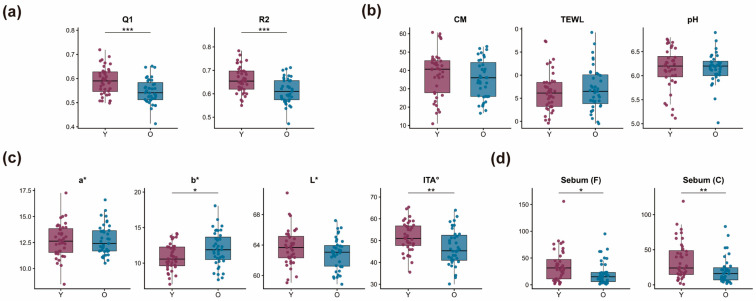
Age-related differences in skin physiological parameters. (**a**) Cheek elasticity: Q1 (recovery efficiency) and R2 (elastic deformation recovery). (**b**) Barrier function: stratum corneum hydration (CM), transepidermal water loss (TEWL), and skin surface pH. (**c**) Skin tone: a* (redness), b* (yellowness), L* (brightness), and ITA° (skin tone index). (**d**) Sebum secretion on the forehead (Sebum[F]) and cheek (Sebum[C]). * *p* < 0.05, ** *p* < 0.01, *** *p* < 0.001. Each dot represents an individual participant; summary statistics are overlaid for each group.

Cheek elasticity parameters (R2 and Q1) were significantly lower in the 29–35 age group (O group) compared to the 22–28 group (Y group) (*p* < 0.001), indicating an age-related decline in viscoelastic performance (Figure 1a). Skin tone analysis showed increased b* and decreased ITA° values in the O group (*p* < 0.05), suggesting darker and less radiant skin compared to the younger group (Figure 1c). No significant differences were observed between groups in skin hydration (CM), transepidermal water loss (TEWL), or surface pH (Figure 1b), indicating overall stability in static barrier parameters. Sebum levels on both the forehead and cheek were significantly higher in the Y group (*p* < 0.05), reflecting a measurable reduction in sebum secretion with age (Figure 1d).

### 3.2. Analysis of Skin Surface Lipid Metabolism Based on Non-Targeted Lipidomics

**Figure 2:**
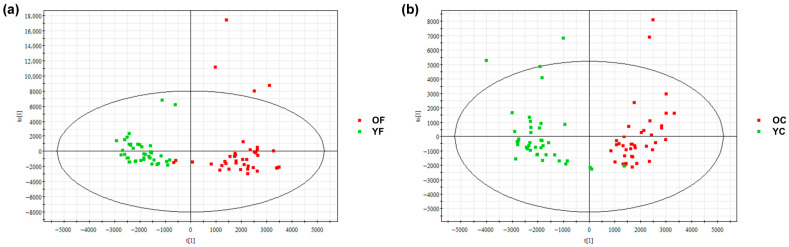
OPLS-DA score plots of skin surface lipid (SSL) profiles in women aged 22–28 (Y) and 29–35 (O). (**a**) Forehead: R^2^Y = 0.85, Q^2^ = 0.74; (**b**) Cheek: R^2^Y = 0.84, Q^2^ = 0.70. Groups are clearly separated. Y: young group; O: older group; F: forehead; C: cheek.

Non-targeted lipidomics of SSLs from the forehead and cheek of combination-skin females (aged 22–28 [Y] and 29–35 [O]) was performed using UPLC-QTof-MS. A total of 1372 lipids were identified and classified into eight LIPID MAPS categories: Sphingolipids (SP), Glycerolipids (GL), Glycerophospholipids (GP), Polyketides (PK), Prenol Lipids (PR), Sterol lipids (ST), Saccharolipids (SL), Fatty Acyls (FA) (Figure 2). OPLS-DA modeling with orthogonal signal correction revealed clear separation between Y and O groups along the t [1] axis (R^2^Y, Q^2^ > 0.5), indicating age- and region-dependent lipidomic profiles and model robustness was assessed by permutation testing (Figure S1).

**Figure 3:**
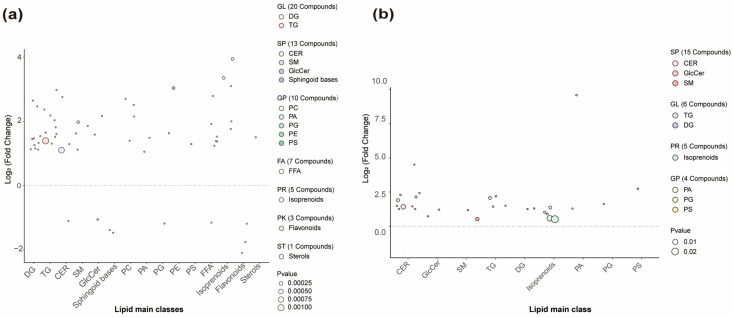
Age-related differential lipids in the forehead and cheek. (**a**) Forehead; (**b**) Cheek. Based on VIP > 1, *p* < 0.05, and fold change > 2 or <0.5, 59 and 30 differential lipids were identified in the forehead and cheek, respectively. The right side of the figure shows lipid class distribution (Class II on x-axis). Lipids above the dashed line show higher abundance in the Y group; those below show lower abundance. DG, diacylglycerols; TG, triacylglycerols; CER, ceramides; SM, sphingomyelins; GlcCer, glucosylceramides; PC, phosphatidylcholines; PA, phosphatidic acids; PG, phosphatidylglycerols; PE, phosphatidylethanolamines; PS, phosphatidylserines; FFA, free fatty acids; GL, glycerolipids; SP, sphingolipids; GP, glycerophospholipids; FA, fatty acyls; PR, prenol lipids; PK, polyketides; ST, sterol lipids; *p* values were obtained from two-group comparisons between Y and O within each region using the tests described in Section 2.5.

Differential lipids between the Y and O groups were identified based on VIP > 1, fold change > 2 or <0.5, and *p* < 0.05. A total of 59 and 30 significant SSL species were detected in the forehead and cheek, respectively, among Chinese women aged 22–28 (Y) and 29–35 (O) with combination skin (Figure 3). These lipids may serve as potential biomarkers of age-related alterations in SSLs. In the forehead, 59 VIP-selected lipids were altered, spanning seven LIPID MAPS primary categories (GL, SP, GP, FA, PR, PK, ST). Within these categories, five VIP-selected lipid subclasses—GlcCer, sphingoid bases, PG, FFA, and flavonoids—showed higher abundance in the O group. Each subclass comprises one or more individual molecular species, and the numbers in brackets on the right side of Figure 3 indicate how many VIP species were assigned to each subclass. Phytosphingosine and C16 sphinganine are two specific sphingoid-base species within the SP category that were identified as VIP lipids in the forehead and exhibited the most pronounced age-related differences within this subclass (Figure 3a). In the cheek, 30 VIP-selected lipids were identified (Figure 3b). Among these, a single ω-O-acyl ceramide species, Cer[EOP], belonging to the ceramide subclass, was selected as a VIP in both facial regions and consistently showed higher abundance in the Y group, indicating that this specific acylceramide is particularly sensitive to early age-related changes in SSL composition.

To visualize species-level patterns of these VIP lipids, hierarchical-clustering heatmaps were generated for the forehead and cheek (Figure S2). In each panel, columns correspond to individual subjects (color-coded as YF vs. OF or YC vs. OC at the top), rows represent the 59 (forehead) or 30 (cheek) VIP molecular species, and the color scale reflects z-score–normalized intensity. In the forehead (Figure S2A), most triacylglycerol (TG) species form a cluster with warmer colors in the YF samples, confirming that sebaceous TGs are relatively enriched in younger skin. By contrast, several sphingomyelin (SM) species and sphingoid bases show warmer colors in OF, indicating higher levels in the older group. Within this latter cluster, the rows labelled “phytosphingosine” and “C16 sphinganine” display noticeably higher signal in OF than in YF, which visually corroborates their identification as O-enriched VIP lipids in the sphingoid-base subclass (Figure 3a). In the cheek (Figure S2B), multiple ceramide and long-chain fatty-acid species show higher intensities in the YC samples, whereas several cholesteryl esters are relatively enriched in OC, consistent with the class-level patterns described above. Together, these heatmaps illustrate that age-related lipid remodeling involves coordinated changes across different lipid classes and highlight phytosphingosine, C16 sphinganine, and Cer[EOP] as representative species with region- and age-dependent abundance. To clarify the analytical hierarchy, VIP-selected lipids in this study refer to individual molecular species identified by the multivariate model, whereas subsequent analyses (e.g., for CER, TG, FFA) summarize aggregated trends at the class or subclass level; therefore, species-level VIP patterns are not expected to match class-level changes in a one-to-one manner.

### 3.3. Correlation of Ceramides with Age and Skin Regional Characteristics

**Figure 4:**
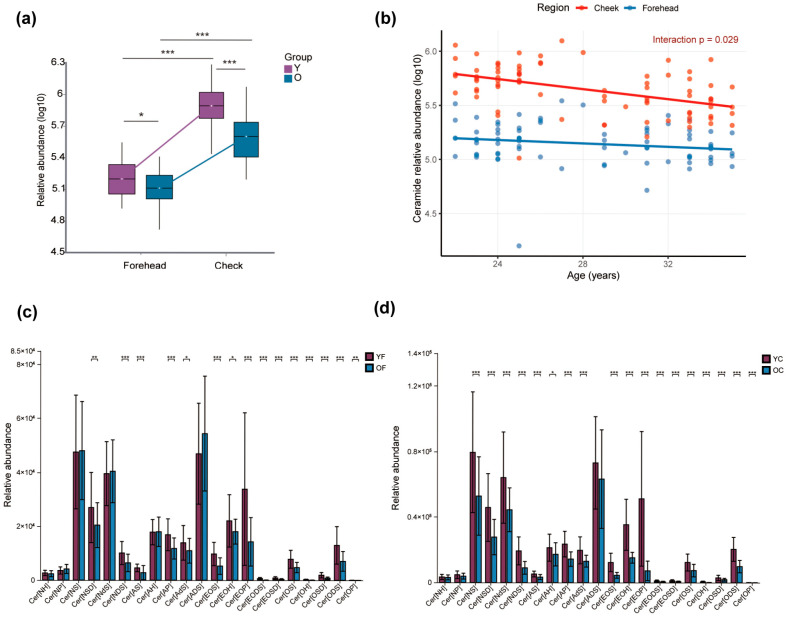
(**a**) Group-wise comparison of total ceramide (CER) levels according to age (Y vs. O) and facial region (forehead vs. cheek). (**b**) Linear mixed-effects regression (random intercept for subject) of CER levels as a function of continuous age and facial region reveals a significant decline with age (β = −0.023, *p* < 0.001), region-specific differences (forehead vs. cheek: β = −0.93, *p* < 0.001), and a significant interaction indicating a steeper age-related decline in the cheek (β = 0.015, *p* = 0.029). Age-dependent alterations in ceramide subclasses across the forehead (**c**) and cheek (**d**). A total of 19 CER subclasses were identified in SSLs, including Cer[NH], Cer[NP], Cer[NS], Cer[EOS], Cer[EOP], and others. * *p* < 0.05, ** *p* < 0.01, *** *p* < 0.001. Region-specific age slopes (β/year) with 95% confidence intervals are summarized in Supplementary Table S2A.

In this section, “CER” denotes the total ceramide class, whereas terms such as Cer[EOP] refer to specific ω-O-acyl ceramide subclasses; subclass-level patterns may therefore differ from the overall class trend. Ceramides (CER), which are essential components of the stratum corneum, play a key role in maintaining skin barrier integrity by promoting lamellar bilayer formation and reducing TEWL. In this study, total CER levels were first compared across facial region and age group (Y vs. O) using categorical analysis (Figure 4a). Both age groups exhibited higher CER levels in the cheek than in the forehead (*p* < 0.001), and within each region the Y group had significantly higher CER levels than the O group (forehead, *p* < 0.05; cheek, *p* < 0.001). This group-wise comparison highlights the spatial heterogeneity of ceramide distribution and indicates that cheek skin is generally more ceramide-rich, but already shows a reduction in the older cohort.

To move beyond this categorical comparison and to characterise the dynamic trend of CER across the entire age range, we next modelled total CER levels as a function of continuous age and facial region (Figure 4b). Linear regression confirmed a significant overall decline in CER concentration with increasing age (β = −0.023, *p* < 0.001) and consistently lower levels on the forehead relative to the cheek (β = −0.93, *p* < 0.001). Importantly, a significant Age × Region interaction (β = 0.015, *p* = 0.029) showed that the age-related reduction in CER was steeper in the cheek than in the forehead, indicating that cheek ceramides are more vulnerable to early age-related loss. Figure 4a visualizes cross-sectional heterogeneity between the four clinical groups (YF, OF, YC, OC), whereas Figure 4b quantifies the age trajectory and demonstrates region-specific differences in the rate of ceramide decline.

Nineteen ceramide (CER) subclasses were identified in SSLs. In the forehead, 14 subclasses (e.g., Cer[AP], Cer[AS]) showed significantly higher abundance in the YF group than in the OF group (*p* < 0.05), while five subclasses (Cer[NH], Cer[NP], Cer[NS], Cer[AH], Cer[ADS]) showed no significant differences (*p* > 0.05) (Figure 4c). Cer[ADS] exhibited a non-significant increase in the OF group, possibly due to individual variability, warranting further validation. In the cheek, 16 CER subclasses were significantly elevated in the YC group compared to the OC group (*p* < 0.05), showing a more marked age effect than in the forehead (Figure 4d). Notably, five O-acyl Cers (Cer[EOP], Cer[EOH], Cer[EOS], Cer[EODS], Cer[EOSD]) were consistently higher in both YF and YC groups. Cer[EOP], with VIP > 1 and *p* < 0.05, was significantly higher in both regions of the Y group, highlighting its potential as a biomarker for age-related changes in facial SSLs metabolism.

### 3.4. Pronounced Age-Related Changes in Cheek Skin Surface Lipid Composition

**Figure 5:**
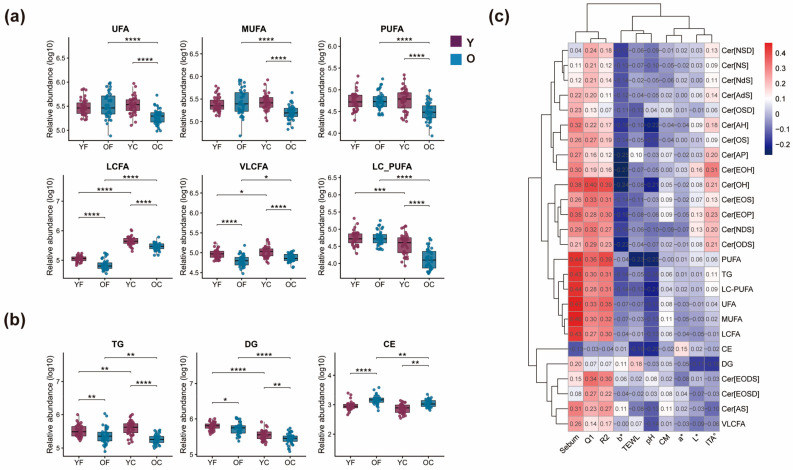
(**a**) Age- and region-dependent variations in FA saturation classes and chain-length categories. UFA, unsaturated fatty acids; MUFA, monounsaturated fatty acids; PUFA, polyunsaturated fatty acids; LCFA, C12–C20, long-chain fatty acids; VLCFA, C > 20, very-long-chain fatty acids; LC-PUFA, long-chain polyunsaturated fatty acids; (**b**) Age-dependent dynamic characteristics of sebaceous gland-derived lipids. Triacylglycerol (TG); diacylglycerol (DG); cholesteryl ester (CE). Statistics: LMM (value ~ age_group × region + (1|subject_id)); planned contrasts were evaluated using emmeans with Holm-adjusted *p* values (*p* < 0.05). (**c**) Heatmap of Pearson’s correlation coefficients between significantly decreased lipids in the cheek region and physiological parameters (sebum, TEWL, CM, pH). * *p* < 0.05, ** *p* < 0.01, *** *p* < 0.001, **** *p* < 0.0001. Effect estimates with 95% confidence intervals for these planned contrasts are reported in Supplementary Table S2B.

Fatty acid (FA) composition by saturation classes and chain-length categories are key determinants of skin barrier integrity and function. Long-chain (C12–C20) and very-long-chain fatty acids (C > 20) support barrier structure and flexibility, while polyunsaturated fatty acids (PUFAs) such as EPA and DHA modulate lipid metabolism and anti-inflammatory responses [16]. In this study, free fatty acid (FFA) profiles were assessed in forehead and cheek regions (Figure 5a). The YC group showed significantly higher levels of unsaturated FAs, including monounsaturated and polyunsaturated species, in the cheek compared to the OC group (*p* < 0.001), with no significant differences in the forehead (*p* > 0.05). Age-related decreases in LCFA and VLCFA levels were observed in both facial regions. Additionally, long-chain PUFAs (LC-PUFAs) declined with age in the cheek but not in the forehead. These results suggest that the cheek region may be more susceptible to age-related lipid alterations and emerging barrier vulnerability due to a more pronounced decline in structural and functional FAs.

Sebum supports skin barrier function, antimicrobial defense, and immune modulation, with alterations implicated in atopic dermatitis (AD) pathogenesis [12]. In this study, age-stratified analysis of sebaceous gland-derived lipids in the forehead and cheek revealed age-related dynamics in TG, diacylglycerol (DG), and CE levels (Figure 5b). A significant decline in TG and DG was observed in the O group compared to the Y group in both regions (*p* < 0.01), suggesting reduced sebaceous lipid synthesis with aging. Conversely, CE levels were significantly elevated in the O group (*p* < 0.001), indicating an opposing trend. These findings suggest differential regulation of sebaceous lipids during aging, with potential implications for skin barrier maintenance and age-associated dermatoses.

In the cheek region, several lipid classes that declined significantly with age also showed specific correlations with physiological parameters (Figure 5c). Together with the observed correlations, these findings suggest that age-related declines in specific ceramide and FFA species may increase susceptibility to barrier impairment and reduced hydration, even though TEWL and CM remained within a similar range between groups in this cohort.

### 3.5. Reaction-Chain Mapping of Differential Cheek Skin Surface Lipids

In this study, “differential lipids” refers to the curated set of annotated lipids reported in Figure 3 (selected using VIP, *p* value, and fold-change). For reaction-chain mapping, we used a broader list of significant lipid species on the cheek (*n* = 334), including partially annotated peaks, to improve network coverage.

**Figure 6:**
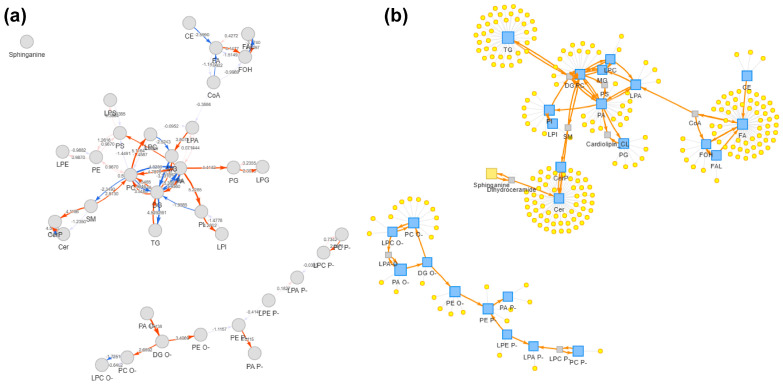
Reaction-chain network mapping of differential cheek skin surface lipid features. (**a**) Pathway activity network built from differential cheek features. Nodes represent mapped lipid classes/entities and edges indicate annotated conversion relationships. Arrowheads indicate the annotated conversion direction (precursor → product). (**b**) Lipid reaction network view showing lipid classes (blue squares) and mapped differential features (yellow circles). The network highlights dominant chains linking glycerolipids and phospholipids. The visualization summarizes co-occurring differences and does not imply metabolic flux or causality. Detailed quantitative results (e.g., pathway scores and chain rankings) are provided in Table S1. Reaction-chain mapping was performed in LipidSig 2.0; differential features were defined using the same exploratory criteria described in the Methods (VIP > 1 with unadjusted *p* < 0.05). PC, phosphatidylcholine; LPC, lysophosphatidylcholine; LPA, lysophosphatidic acid; PA, phosphatidic acid; PI, phosphatidylinositol; LPI, lysophosphatidylinositol; PG, phosphatidylglycerol; LPG, lysophosphatidylglycerol.

Reaction-chain mapping was applied to the differential cheek skin surface lipid (SSL) features using LipidSig 2.0. A total of 334 differential features were identified for the cheek using the exploratory criteria described above. The top-ranked conversion chains mainly connected glycerolipids to phospholipids, with the highest-scoring chain being TG → DG → PC → LPC → LPA → PA → PI → LPI (pathway score = 8.898), together with closely related variants involving PI/LPI and PG/LPG (Table S1). Consistent with this network, the differential feature list showed a clear directional pattern: most TG and DG features were lower in the older group, whereas phospholipid features were more frequently higher. In parallel, ceramides were more often lower in the older group, while glucosylceramides were predominantly higher. Two cholesterol ester features were also higher in the older group (*p* < 0.05). The network view (Figure 6) summarizes coordinated class-level shifts among differential cheek SSL features. Forehead network visualizations, included as a regional comparison, are shown in the Supplementary Materials (Figures S3 and S4).

## 4. Discussion

Motivated by the Traditional Chinese Medicine (TCM) concept of 7-year physiological cycles as a cultural hypothesis driver, we focused on the late-twenties window and integrated facial biophysics with untargeted skin surface lipidomics in women with combination skin. Skin surface lipids (SSLs) comprise sebaceous and intercellular components and contribute to barrier homeostasis and antimicrobial defense [9]. We observed stable conventional barrier readouts (TEWL, hydration, pH) alongside cheek-predominant molecular remodeling in surface lipids, suggesting that early lipid reorganization may occur before overt changes in standard biophysical indices become detectable.

Cheek elasticity (Q1, R2) was lower in the older group, consistent with early changes in dermal matrix homeostasis and age-related shifts in skin mechanical recovery. Reduced sebum secretion in the older group may further contribute to perceived dryness, fatigue, and loss of firmness in combination skin [17,18].

The increase in b* and decrease in ITA° in the older group indicate early dullness and pigment accumulation, which aligns with reports linking photoaging-related oxidative stress and endocrine changes with altered pigmentation trajectories after the late twenties [19,20]. Taken together, the physiological data indicate that, even within a relatively narrow age range, the cheek already shows a combination of reduced elasticity, lower sebum output and subtle pigment changes, supporting its reputation as a “fragile” zone in combination skin.

TEWL, hydration (CM) and surface pH did not differ between age groups, indicating that static barrier readouts may remain insensitive within a narrow early-adult age range. This does not exclude ongoing remodeling, because gradual changes in lipid composition and organization can precede detectable shifts in TEWL, and barrier recovery can be modulated by topical lipid ratios [21] and cellular processes such as autophagy that shape epidermal lipid composition [22]. Together, these observations support that lipidomics provides complementary information beyond TEWL/CM/pH measures and can reveal early adaptive changes in barrier-related lipid metabolism [23,24].

To further contextualize the differential cheek SSL features, we performed reaction-chain mapping using LipidSig 2.0 (Figure 6; Table S1). The top-ranked chains mainly linked sebaceous glycerolipids (TG/DG) with downstream phospholipid classes (TG → DG → PC/LPC → LPA/PA → PI/LPI), consistent with the feature-level directionality observed in our dataset (lower TG/DG but more frequently higher phospholipid features in the older group). This network summarizes co-varying lipid features and serves as a hypothesis-generating tool, not proof of altered metabolic flux. Forehead networks are provided in the Supplementary Materials for regional comparison.

Ceramides (CER) emerged as a sensitive class in our dataset. Total CER levels were higher on the cheek than the forehead but declined with age in both regions, with a steeper reduction on the cheek. Several ω-O-acyl ceramide subclasses (e.g., Cer[EOP]/Cer[EOS]/Cer[EOH]) were consistently higher in the younger group, consistent with their structural role in long-periodicity lamellae and barrier cohesion [25,26,27]. Although we did not quantify enzyme expression or detailed chain-length distributions, the selective reduction of acylceramide-related features on the cheek suggests early structural remodeling that may precede overt TEWL changes [28,29,30,31].

We also observed coordinated changes in sphingoid bases and other sphingolipid-related species. On the forehead, VIP-based analysis identified phytosphingosine and C16 sphinganine as representative sphingoid bases with higher abundance in the O group, and their enrichment was clearly visualized in the forehead heatmap. Sphingoid bases and their phosphorylated metabolites, such as sphingosine-1-phosphate, are known to participate in keratinocyte differentiation, inflammatory signaling and barrier repair [32,33,34]. Given the cross-sectional design, these patterns should be interpreted as associations that may reflect compensatory responses to cumulative stress rather than mechanistic causality.

Changes in fatty acids (FAs) and glycerolipids provide further insight into region-specific susceptibility. Long-chain and very-long-chain FFAs are structural components of the stratum corneum lipid matrix and are crucial for maintaining tight packing and low permeability [35]. Experimental models show that shortening of FA chain length weakens lipid packing and increases permeability, whereas enrichment of long-chain FAs reduces TEWL and improves barrier robustness [29,31]. Polyunsaturated FAs additionally modulate inflammation and epidermal homeostasis through lipid mediators and genomic signaling [25,36]. In our cohort, age-related declines in long-chain and polyunsaturated FFAs were more pronounced on the cheek than on the forehead, and several of these FAs, together with specific CER subclasses and glycerolipids (TG, DG), were positively correlated with sebum and hydration and negatively correlated with TEWL. These correlations support a model in which cheek-specific loss of structural FAs and sebaceous glycerolipids contributes to reduced hydration resilience and an increased tendency toward micro-barrier impairment, even when group-level TEWL differences are not yet apparent.

Free fatty acids further influence barrier behavior in a chain-length- and structure-dependent manner. In skin lipid models, enrichment of short-chain or more disordered fatty acids loosens lipid packing and increases permeability, whereas long-chain saturated and monounsaturated species enhance barrier capability; by contrast, highly unsaturated species participate in both pro- and anti-inflammatory pathways and in the resolution of inflammation [37]. Clinical and ex vivo work has also linked alterations in sebum composition—including changes in the relative abundance of triglycerides, free fatty acids, squalene and its oxidation products, and lipophilic antioxidants such as vitamin E, to acne and other conditions characterized by oxidative stress and microinflammation [38]. This mechanistic background provides a plausible link between the age-related decline in cheek long-chain FFAs and sebaceous glycerolipids observed in our cohort and a reduced capacity to buffer environmental and inflammatory insults over time.

Translationally, the cheek-predominant reduction in acylceramide- and long-chain fatty-acid-related features suggests that lipid-supportive strategies may be more relevant for the U-zone in combination skin during early adulthood. Formulation concepts to be evaluated in future work include barrier-lipid replenishment approaches (ceramide, cholesterol, free fatty acids in lamellar systems), and targeted support of acylceramide-associated structures (e.g., providing appropriate linoleate-containing lipid precursors), alongside standard hydration support. These implications remain hypothesis-generating and require targeted lipid quantification and intervention studies.

The regional contrast between cheek and forehead is likely multifactorial. The forehead has a higher density of sebaceous glands and a thicker sebum film, which may buffer early lipid depletion and environmental insults [10,17]. The cheeks, by contrast, receive less sebaceous input and are more exposed to ambient temperature fluctuations, UV radiation and friction, making them more prone to lipid loss and microdamage. Emerging work also suggests that facial subregions harbor distinct microbiota and microenvironments, which may interact with local lipid metabolism and inflammation; further integration of lipidomics and microbiome profiling will be needed to disentangle these contributions in future studies.

Finally, our findings resonate conceptually with the TCM description of a functional turning point around age 28, but they do not demonstrate that reaching this age “causes” lipid remodeling. Instead, our cross-sectional comparison of women in their early and early-mid thirties identifies an association between the late-twenties window and the onset of region-specific lipid shifts, especially on the cheeks. These empirical patterns offer molecular context for the traditional notion of a 28-year threshold but should be viewed as observational support rather than mechanistic proof. Longitudinal studies tracking individual trajectories across this age range will be required to determine whether the observed lipid changes represent a true inflection point or part of a more gradual continuum.

This study has several limitations. First, its cross-sectional design and modest sample size limit causal inference and generalizability. Potential confounders (e.g., UV exposure, sleep, menstrual/hormonal status, habitual diet, and skincare routines) were not systematically quantified in this cohort; therefore, the reported associations are unadjusted and should be interpreted as exploratory. Future studies with standardized exposure assessment and multivariable adjustment are needed to confirm causality and improve generalizability. Second, we did not directly assess stratum corneum microstructure, microbiota, or transcriptomic changes, which constrains mechanistic interpretation of the lipid shifts. Third, the cohort consisted of healthy Chinese women with combination skin, so extrapolation to other ethnicities, sexes or skin types should be cautious. Despite these limitations, the integration of regional lipidomics with physiological measurements provides an early molecular map of age-associated barrier adaptation and highlights cheek-targeted ceramide and fatty-acid support as a promising strategy for precision skincare around the late-twenties transition.

## 5. Conclusions

In this cross-sectional study of Chinese women with combination skin, we identified region- and age-associated differences in skin surface lipids between the 22–28 and 29–35 year groups, most notably lower acylceramides, long-chain fatty acids and sebaceous glycerolipids on the cheeks of the older group, while TEWL, hydration and pH remained broadly comparable. These findings suggest early lipid remodeling and emerging barrier vulnerability rather than overt dysfunction, and are conceptually compatible with, but do not prove, the notion of a “threshold” phase of skin aging around the late twenties. Within these limits, cheek-focused lipid changes may serve as sensitive markers of incipient aging in combination skin and provide a rationale for region-specific, lipid-supportive skincare strategies.

## Data Availability

The data generated during the current study are available from the corresponding author on reasonable request.

## Supplementary Materials

The following supporting information can be downloaded at: https://www.mdpi.com/article/10.3390/metabo16010073/s1, Figure S1: Permutation test results; Figure S2: Age-related differential lipids in the forehead and cheek; Figure S3: Reaction-chain network of differential forehead skin surface lipid features; Figure S4: Forehead lipid conversion network visualization; Table S1: pathway score; Table S2: LMM Effect Estimates.
