# Supplementary material for: Region-Specific Lipid Alterations Around the 28-Year Transition as Early Indicators of Skin Aging
Source: Metabolites. 2026 Jan 13;16(1):73. doi: 10.3390/metabo16010073 (PMC12843811; doi:10.3390/metabo16010073)
Supplement: Supplementary file 1 [file metabolites-16-00073-s001.zip › metabolites-4088909-supplementary.pdf]

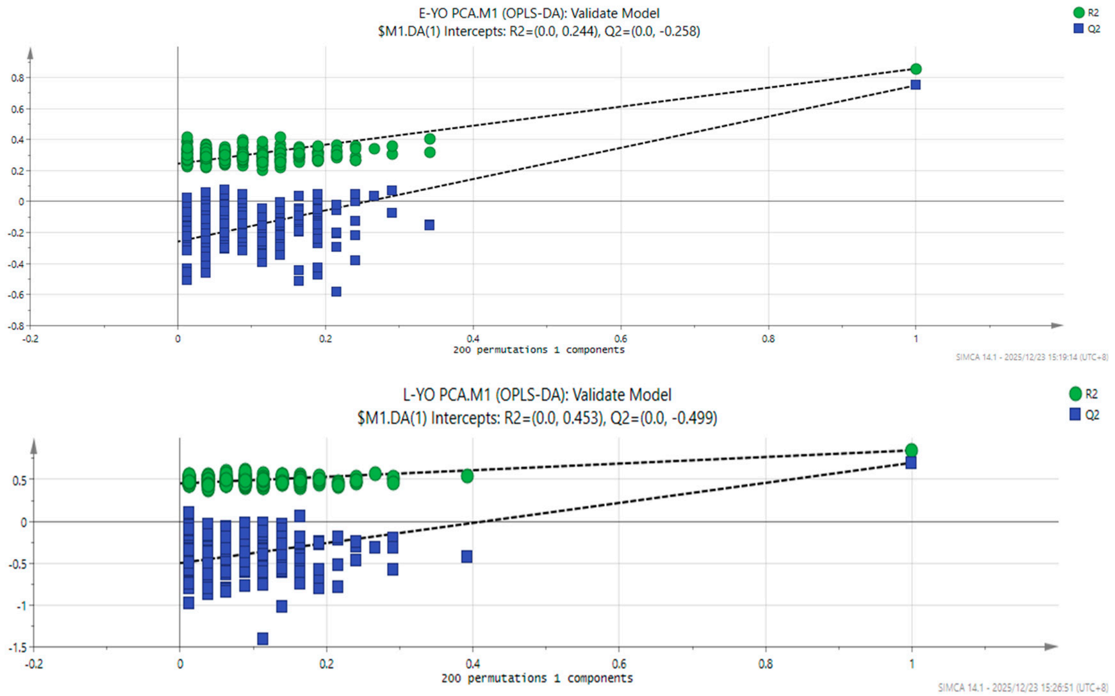

Figure S1. Permutation testing.

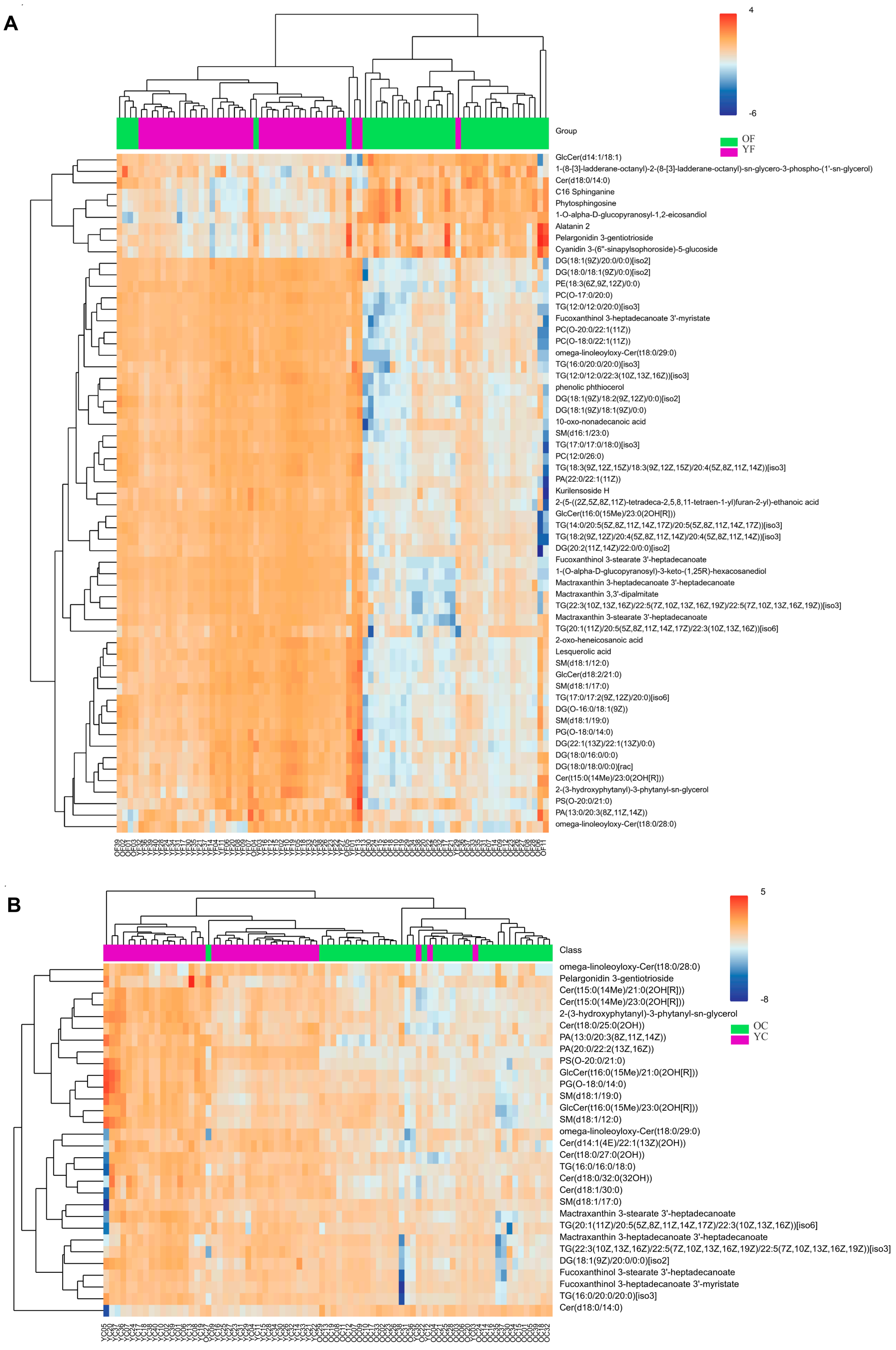

Figure S2. Heatmap of age-related differential skin surface lipids (SSL) in the forehead and cheek. Relative abundance values were z-score normalized within each lipid feature across all samples (z = (x - mean)/SD) prior to visualization. The color bar indicates z-score values (blue: lower relative abundance; red: higher relative abundance).

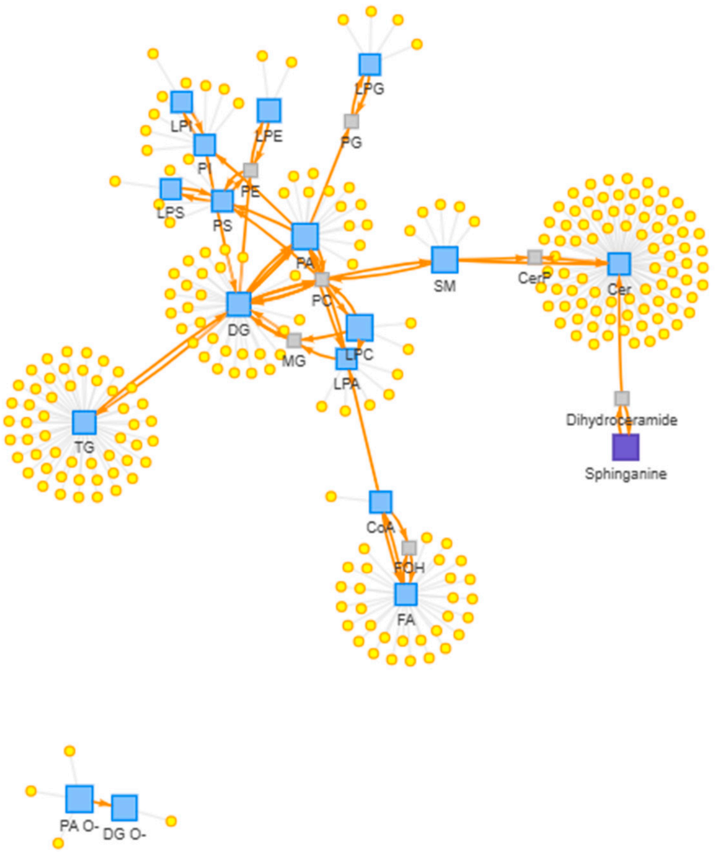

Figure S3. Reaction-chain network of differential forehead skin surface lipid (SSL) features generated using LipidSig 2.0. Nodes represent differential lipid features and edges indicate annotated conversion relationships based on lipid class reaction rules. This network is intended for hypothesis generation and pathway-style interpretation, and does not provide direct evidence of metabolic flux or causality.

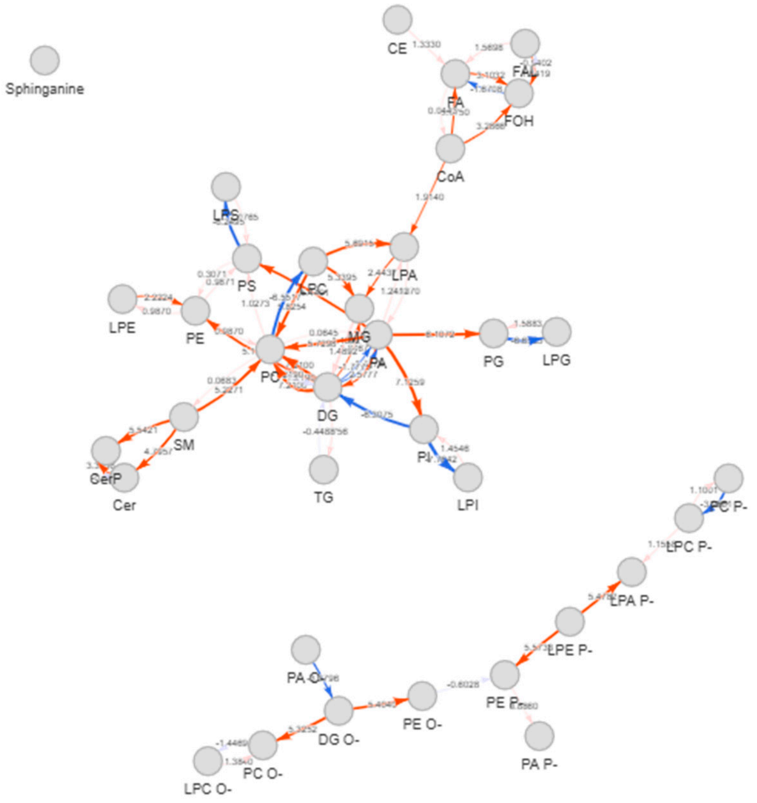

Figure S4. Forehead lipid conversion network visualization (LipidSig 2.0). A complementary visualization of the differential forehead SSL reaction-chain mapping is provided for comparison. The inferred conversion relationships are annotation-based and should be interpreted as hypothesis-generating; they do not imply metabolic flux or causality.

Table S1. LipidSig 2.0 reaction-chain mapping of differential cheek skin surface lipid (SSL) features: ranked conversion chains and pathway scores. (provided as an Excel workbook "Table S1.xlsx", containing multiple sheets).

Table S2. Effect estimates (with 95% confidence intervals) for planned contrasts in Fig. 5 and region-specific age slopes in Fig. 4b.

Table S2A. Region-specific age slopes for Fig. 4b (Kenward–Roger df).

Region	Age slope (β/year)	95% CI	p value
Cheek	-0.02305	-0.03290 to -0.01322	<0.0001
Forehead	-0.00769	-0.01750 to 0.00214	0.1235

Table S2B. Planned contrasts for Fig. 5 (LMM on log10-transformed values).

metric	comparison	Est (log10)	95% CI (log10)	P_adj (Holm)	Ratio (10^est)
UFA	Age effect (Forehead)	-0.043	-0.155 to 0.068	0.365	0.905 (0.700–1.170)
UFA	Age effect (Cheek)	0.241	0.130 to 0.353	<0.0001	1.743 (1.348–2.254)
UFA	Region effect (Young)	-0.043	-0.124 to 0.039	0.365	0.906 (0.751–1.093)
UFA	Region effect (Older)	0.242	0.160 to 0.324	<0.0001	1.746 (1.444–2.110)
MUFA	Age effect (Forehead)	-0.050	-0.166 to 0.066	0.466	0.891 (0.682–1.165)
MUFA	Age effect (Cheek)	0.230	0.114 to 0.346	<0.0001	1.698 (1.299–2.219)
MUFA	Region effect (Young)	-0.041	-0.129 to 0.046	0.466	0.910 (0.744–1.113)
MUFA	Region effect (Older)	0.239	0.150 to 0.327	<0.0001	1.732 (1.413–2.124)
PUFA	Age effect (Forehead)	-0.007	-0.134 to 0.121	0.890	0.984 (0.734–1.320)
PUFA	Age effect	0.280	0.152 to	<0.0001	1.904

	(Cheek)		0.407		(1.420–2.554)
PUFA	Region effect (Young)	-0.026	-0.111 to 0.059	0.880	0.942 (0.775–1.146)
PUFA	Region effect (Older)	0.261	0.175 to 0.347	<0.0001	1.823 (1.495–2.223)
LCFA	Age effect (Forehead)	0.205	0.127 to 0.283	<0.0001	1.604 (1.340–1.920)
LCFA	Age effect (Cheek)	0.192	0.114 to 0.270	<0.0001	1.556 (1.300–1.863)
LCFA	Region effect (Young)	-0.604	-0.680 to -0.528	<0.0001	0.249 (0.209–0.297)
LCFA	Region effect (Older)	-0.617	-0.694 to -0.540	<0.0001	0.242 (0.202–0.288)
VLCFA	Age effect (Forehead)	0.164	0.094 to 0.235	<0.0001	1.460 (1.242–1.717)
VLCFA	Age effect (Cheek)	0.146	0.075 to 0.216	<0.0001	1.398 (1.189–1.644)
VLCFA	Region effect (Young)	-0.055	-0.121 to 0.010	0.035	0.880 (0.756–1.024)
VLCFA	Region effect (Older)	-0.074	-0.141 to -0.007	0.012	0.843 (0.723–0.983)
LC_PUFA	Age effect (Forehead)	-0.002	-0.148 to 0.144	0.973	0.995 (0.711–1.393)
LC_PUFA	Age effect (Cheek)	0.440	0.294 to 0.586	<0.0001	2.755 (1.969–3.855)
LC_PUFA	Region effect (Young)	0.176	0.062 to 0.290	0.0003	1.499 (1.154–1.948)
LC_PUFA	Region effect (Older)	0.618	0.503 to 0.733	<0.0001	4.149 (3.182–5.409)
TG	Age effect	0.136	0.025 to	0.007	1.367

	(Forehead)		0.247		(1.059–1.764)
TG	Age effect (Cheek)	0.334	0.223 to 0.444	<0.0001	2.156 (1.670–2.783)
TG	Region effect (Young)	-0.092	-0.177 to -0.007	0.007	0.809 (0.665–0.984)
TG	Region effect (Older)	0.106	0.020 to 0.192	0.007	1.276 (1.046–1.556)
DG	Age effect (Forehead)	0.069	-0.012 to 0.150	0.033	1.172 (0.972–1.413)
DG	Age effect (Cheek)	0.097	0.016 to 0.179	0.006	1.251 (1.038–1.509)
DG	Region effect (Young)	0.256	0.174 to 0.338	<0.0001	1.803 (1.494–2.176)
DG	Region effect (Older)	0.284	0.202 to 0.367	<0.0001	1.925 (1.591–2.329)
CE	Age effect (Forehead)	-0.237	-0.364 to -0.110	<0.0001	0.579 (0.432–0.776)
CE	Age effect (Cheek)	-0.175	-0.302 to -0.048	0.002	0.668 (0.498–0.895)
CE	Region effect (Young)	0.059	-0.046 to 0.163	0.156	1.144 (0.899–1.456)
CE	Region effect (Older)	0.120	0.014 to 0.226	0.010	1.319 (1.034–1.684)
